# Clustering of quantitative CT features identifies HCC subtypes with distinct prognosis and immune signatures

**DOI:** 10.1186/s41747-026-00730-1

**Published:** 2026-05-27

**Authors:** Shuang Wu, Fang Peng, Jiexing Huang, Lingrui Xu, Xiaoyue Zhang, Yong Bao, Yong Chen

**Affiliations:** 1 Department of Radiation Oncology, The First Affiliated Hospital, Sun Yat-sen University, Guangzhou, China; 2 Cancer Center, The First Affiliated Hospital, Sun Yat-sen University, Guangzhou, China

**Keywords:** Cluster analysis, Hepatocellular carcinoma, Immune checkpoint inhibitors, Tumor microenvironment, Radiomics

## Abstract

**Objective:**

There is still a lack of widely applicable biomarkers for immunotherapy in hepatocellular carcinoma (HCC). We aim to identify subtypes using computed tomography (CT) imaging features of HCC and assess their value in predicting prognosis and the effectiveness of immunotherapy.

**Materials and methods:**

We used unsupervised consensus clustering based on quantitative contrast-enhanced CT features to identify imaging subtypes and investigated their value in predicting prognosis and the effectiveness of immunotherapy in the discovery (*n* = 103) and immunotherapy-treated (*n* = 110) validation cohort. We also developed a gene-based classifier for imaging subtypes and tested their prognostic and biological relevance in two additional gene validation cohorts with publicly available gene expression data but without imaging data (*n* = 551).

**Results:**

The imaging subtypes demonstrated significant correlations with overall survival across all cohorts (*p* = 0.002, *p* < 0.001) and effectively predicted immunotherapy outcomes, with 1-year progression-free survival rates varying significantly (*p* < 0.001). Imaging subtypes 1 and 3 with favorable and intermediate immunotherapy responses showed significant activation of B-cells, lymphocyte-mediated immunity, and immune response (adjusted *p*-value ≤ 0.047, false discovery rate ≤ 0.043). Imaging subtype 2 with poor immunotherapy responses displayed the least activated CD8+ T cells, the lowest cytolytic activity, and the highest infiltration of neutrophil and regulatory T cells. Additionally, type I and II interferon responses were significantly downregulated in imaging subtype 2.

**Conclusion:**

Unsupervised clustering of CT imaging features identified subtypes with significantly distinct prognosis and immune signatures. The imaging subtypes can serve as a marker for immunotherapy in HCC.

**Relevance statement:**

The imaging subtypes identified in this study demonstrate significant clinical relevance by providing a non-invasive approach to predict prognosis and immunotherapy response for HCC.

**Key Points:**

- We identified three novel imaging subtypes using unsupervised consensus clustering of quantitative CT imaging features for HCC.
- Imaging subtypes were independent predictors of overall survival and the effectiveness of immunotherapy for HCC.
- The imaging subtypes can be used as a marker for immunotherapy in HCC.

**Graphical Abstract:**

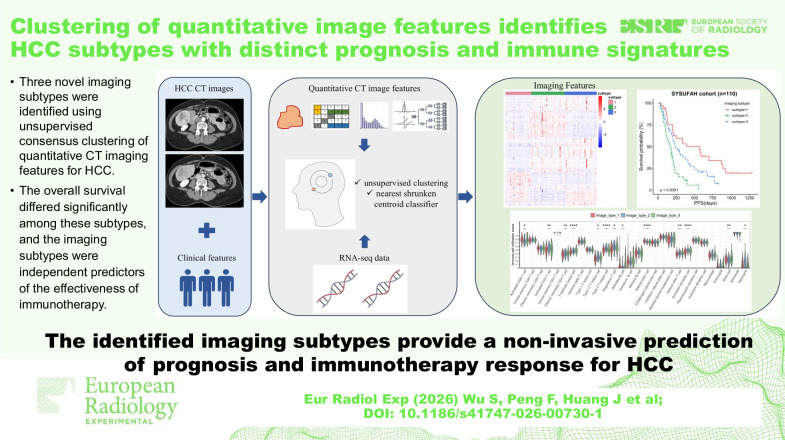

## Background

Liver cancer poses a substantial global health challenge, being the fifth and seventh most common cause of cancer-related death in men and women, respectively [1, 2]. Hepatocellular carcinoma (HCC), the predominant histological subtype of primary liver cancer, carries a poor prognosis, as evidenced by its low 5-year OS rate [3]. While immune checkpoint inhibitors (ICIs) have been approved by the USA Food and Drug Administration for HCC treatment, their efficacy remains limited, with objective response rates of approximately 20% for monotherapy and 30% for combination regimens [4–6]. This highlights the urgent need for identifying predictive markers to optimize patient selection for ICIs-based treatment strategies.

Several biomarkers—including tumor-infiltrating immune cells, programmed death-ligand 1 (PD-L1) expression, tumor mutational burden (TMB), molecular signatures, and blood-based markers—have been explored for their ability to predict response to ICIs in HCC [7]. However, the clinical application of these biomarkers has been limited by issues such as tumor heterogeneity, methodological variations in detection techniques, small sample sizes, and inconsistent results across studies. Additionally, the analysis of these biomarkers relies on tumor tissue samples, which are not always available, given that HCC can be diagnosed through imaging alone [8]. Therefore, there is a critical need to develop non-invasive and reproducible biomarkers to optimize clinical decision-making in immunotherapy.

Emerging evidence indicates that radiomic features extracted from computed tomography (CT) imaging can serve as predictors for the macrotrabecular-massive subtype [9, 10], microvascular invasion [11, 12], and response to lenvatinib treatment in HCC [13]. Several studies have also explored the correlation between CT imaging features and the efficacy of ICIs. Three independent cohorts (*n* = 58, *n* = 55, and *n* = 151) demonstrated that CT-based radiomic signatures could serve as potential biomarkers for predicting response to ICIs [14–16]. In another study, imaging features were indirectly linked to immunotherapy outcomes through a predictive model developed for neutrophil extracellular traps [17]. Additionally, a study comprising 105 patients employed CT radiomics to estimate PD-1 expression levels and prognosis in individuals with HCC. It should be noted that the enrolled patients did not receive PD-1 inhibitor therapy but were administered sorafenib [18]. These studies, however, were limited by small sample sizes and a lack of large-scale validation. Moreover, the biological mechanisms that connect imaging phenotypes to immunotherapy efficacy remain incompletely elucidated.

In this study, we applied quantitative CT radiomics to identify novel HCC subtypes using unsupervised consensus clustering. The imaging-defined subtypes were validated in independent cohorts for their ability to predict both prognostic outcomes and response to immunotherapy. Furthermore, we characterized the immunogenomic profiles potentially associated with these subtypes by establishing a comprehensive imaging-genomic correlation map. Our results offer clinically applicable imaging biomarkers that may help identify HCC patients most likely to benefit from immunotherapy.

## Methods

**Fig. 1:**
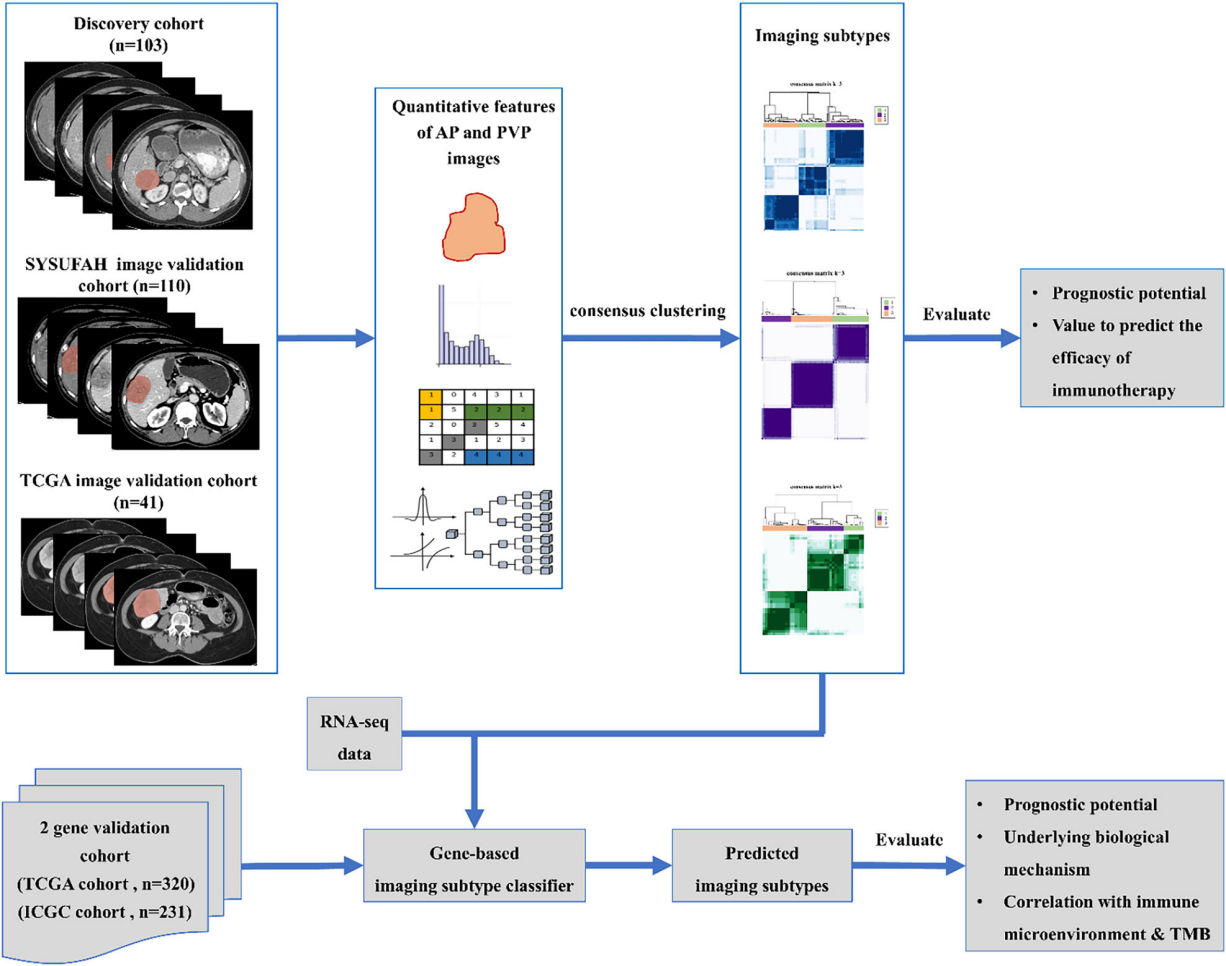
Analysis workflow. AP, Arterial phase; ICGC, International Cancer Genome Consortium; PVP, Portal venous phase; SYSUFAH, First Affiliated Hospital of Sun Yat-sen University; TCGA, The Cancer Genome Atlas; TMB, Tumor mutation burden

This study was approved by our institutional review board; written informed consent was waived due to the retrospective nature of the study. The analysis workflow is shown in Fig. 1.

### Patient cohorts

The discovery cohort was collected at the MD Anderson Cancer Center institutional database between November 2002 and June 2012 [19]. In short, 105 patients were diagnosed with HCC and had available CT images obtained before treatment. The arterial phase and portal venous phase CT images showed no apparent artifacts. Two patients were excluded due to the tumor not being fully displayed or lacking a portal venous phase image, resulting in 103 patients eligible for the study.

The First Affiliated Hospital of Sun Yat-sen University (SYSUFAH) image validation cohort was collected from January 1, 2020, to June 30, 2024, at our center. The inclusion criteria were as follows: (1) a confirmed diagnosis of HCC by histopathology or imaging; (2) contrast-enhanced CT scans performed within 1 month prior to treatment initiation; (3) availability of images from both the arterial and portal venous phases; (4) the presence of at least one measurable lesion on CT (Response Evaluation Criteria in Solid Tumors‒RECIST version 1.1); (5) received consecutive treatment with anti-PD-L1/PD-1 monoclonal antibodies as per their physician’s protocol; and (6) with complete clinical data. Exclusion criteria comprised: (1) a concurrent diagnosis of any other malignancy; (2) loss to follow-up; and (3) pregnancy. The study ultimately analyzed 110 patients. Progression-free survival (PFS) was measured from immunotherapy initiation to disease progression or last follow-up, and overall survival (OS) to death or last follow-up.

The Cancer Genome Atlas (TCGA)-Liver Hepatocellular Carcinoma (LIHC) dataset comprises 371 patients with liver cancer, including 369 with primary tumors and 2 with recurrent tumors [20]. Cases with primary tumors were included in the study. Among the 369 cases, 41 cases of hepatocellular carcinoma having imaging data were defined as the TCGA-LIHC image validation cohort. Among the remaining 328 cases, 7 cases of mixed hepatocellular carcinoma and cholangiocarcinoma, as well as 1 case of clear cell adenocarcinoma, were excluded. Finally, 320 HCC cases having RNA-seq data were defined as the TCGA-LIHC gene validation cohort.

LIRI-JP (Liver cancer-RIKEN, JP) project involved tumor samples from 231 patients with HCC in the Japanese population who were infected with the hepatitis B virus or hepatitis C virus [21]. This dataset was referred to as the International Cancer Genome Consortium‒ICGC-LIRI-JP gene validation cohort in the study.

### Image segmentation and feature extraction

Image segmentation and radiomics feature extraction were performed using 3D Slicer (version 5.3.0). In the discovery cohort, the tumor segmentations were downloaded from the TGCA database [19]. In the TCGA-LIHC and SYSUFAH image validation cohorts, the area confirmed as tumor tissue on the CT image was outlined after careful comparison with peritumoral tissue. When there is uncertainty in identifying the tumor region, two experienced doctors reached a consensus. We calculated the DICE coefficient to evaluate inter-reader reliability. The average Dice similarity coefficient was 0.892. Next, voxels in each CT image volume were resampled to an isotropic voxel size of 1 × 1 × 1 mm^3^. For the discretization of the image gray levels, the bin width was set to 25. Finally, 1,702 quantitative radiomics features were extracted [22] (Supplementary Table S1). The quantitative radiomics features were normalized using z-score normalization.

### Imaging subtype discovery and validation

Unsupervised consensus clustering [23] was used to identify intrinsic imaging subtypes. The partition-around-medoids clustering algorithm with the Spearman distance metric was repeated for 1,000 repetitions by resampling 80% of items. The cluster number was set from 2 to 10 to determine the optimal number of clusters. Finally, we identified three imaging subtypes. We assessed the relationship between these subtypes and the clinical characteristics of HCC through chi-square tests or analysis of variance (ANOVA), with a *p*-value < 0.05 considered statistically significant. For multiple comparisons, we applied the Benjamini–Hochberg correction. To assess the consistency of imaging subtypes between the validation and discovery cohorts, patient-level imaging features were visualized with standardized heatmaps, and the IGP (in-group proportion) statistic was calculated [24].

To further compare the differences in imaging features between the discovery cohort and the validation cohort, and to demonstrate the high consistency of imaging subtypes obtained through clustering in both the discovery and validation cohorts, we performed principal component analysis on the imaging features of the discovery and validation cohorts, respectively. Principal component analysis is a statistical method that uses linear transformation to convert a set of potentially correlated variables into a new set of linearly uncorrelated variables (called principal components), while preserving as much information from the original data as possible [25]. After conducting principal component analysis, we compared the differences in principal component features between the discovery cohort and the validation cohort.

### Prognostic significance of imaging subtypes

We used the Kaplan–Meier analysis and log-rank test to evaluate the prognostic ability of imaging subtypes to predict OS. To further explore the role of imaging subtypes in predicting the effectiveness of immunotherapy, we used the Cox proportional hazard model to construct survival models for predicting PFS and OS in the SYSUFAH cohort. In order to investigate whether imaging subtypes can serve as prognostic markers in the two gene validation cohorts, we developed a gene-based imaging subtype classifier using imaging subtype labels and RNA-seq data in the TCGA-LIHC image validation cohort. Specifically, we filtered genes expressed in at least 50% of samples in both TCGA and ICGC cohorts for subsequent analysis. We then used ANOVA or Kruskal’s test along with the Cox proportional hazard model to identify genes significantly associated with imaging subtypes and survival. Finally, 40 genes were selected. Based on selected genes, we trained a nearest shrunken centroid classifier [26] and performed 10-fold cross-validation. The trained classifier was used to define imaging subtypes for each patient in the ICGC-LIRI-JP as well as the TCGA-LIHC gene validation cohort, and the prognostic value of imaging subtypes was assessed.

### Biological mechanisms underlying the imaging subtypes

The differential gene expression analysis was conducted using the R package “DESeq2” [27]. Since imaging subtype 2 had the worst prognosis, its gene expression was set as the baseline. Significantly differentially expressed genes were defined as those with FDR (false discovery rate) < 0.05 and |fold change| > 2. Gene set enrichment analysis (GSEA) was then conducted to identify molecular pathways significantly associated with imaging subtypes *via* the “clusterProfiler” package [28]. The “C5: ontology gene sets” from the MsigDB database was downloaded as the background gene set. Pathways with adjusted *p* < 0.05 and FDR < 0.05 were identified as significantly enriched.

### Correlation of imaging subtypes with immune microenvironment and TMB

To evaluate the correlation between tumor immune microenvironment and imaging subtypes, single-sample GSEA was performed to calculate the enrichment scores of 28 immune cells and 13 immune signatures through the R package “GSVA” [29]. Simple nucleotide variation data were downloaded, and then TMB was calculated *via* the R package “maftools” [30]. Subsequently, the association between TMB and imaging subtypes was further evaluated.

### Statistical analysis

The z-score normalization, consensus clustering, nearest shrunken centroid classifier training, ANOVA test, Kruskal’s test, chi-square (Fisher) test, IGP calculation, Kaplan–Meier analysis, log-rank test, the Cox proportional hazard model, differential gene expression analysis, GSEA, and single-sample GSEA were all performed in R (version 4.2.3). We applied the Benjamini–Hochberg method to control FDR. A two-sided p 0.05 was considered statistically significant for all statistical tests.

## Results

### Three imaging subtypes were identified and significantly associated with OS

**Fig. 2:**
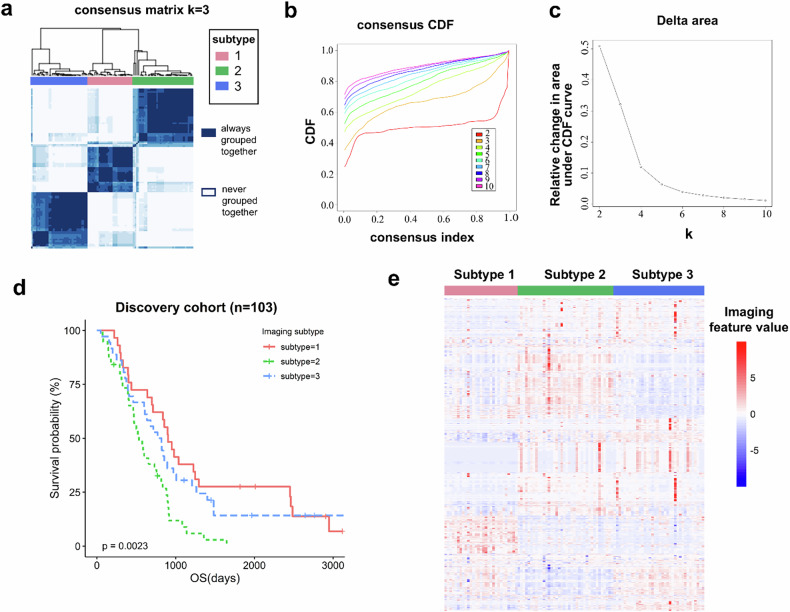
Three clusters were identified and significantly associated with OS in the discovery cohort. We identified 3 imaging subtypes through unsupervised consensus clustering. **a** A heatmap representing consensus matrices. In the heatmap, both rows and columns represent subjects, and consensus values range from 0 (white, never clustered together) to 1 (dark, always clustered together). **b** Consensus clustering utilized the CDF curve to identify the optimal number of clusters. **c** The relative change in the area under the CDF curve when the number of clusters changed from k to k + 1. **d** A significant difference in OS was observed among imaging subtypes. **e** Imaging features were visualized with a heatmap. Heatmap columns represent individual patients (grouped according to the clusters discovered by unsupervised consensus clustering), and each row is an imaging feature. *p*-values were from the Log-rank test. A two-sided *p* < 0.05 was considered statistically significant. CDF, Cumulative distribution function; OS, Overall survival

Using consensus clustering, we identified three imaging subtypes in the discovery cohort (Fig. 2a–c). The distribution of patients across these subtypes was as follows: 29 (28.2%) in imaging subtype 1, 38 (36.9%) in imaging subtype 2, and 36 (34.9%) in imaging subtype 3. No significant associations were observed between the imaging subtypes and established clinical features of HCC, including age, evidence of cirrhosis, Child-Pugh score, vascular invasion, distant metastasis, lymph nodes metastasis, portal vein thrombosis, alpha-fetoprotein, and BCLC (Barcelona Clinic Liver Cancer) stage (Table 1). However, a significant difference in OS was noted among the three subtypes (*p* = 0.0023; Fig. 2d). The 2-year OS rates for imaging subtypes 1, 2, and 3 were 62.07%, 38.03%, and 52.78%, respectively. The imaging features for each subtype are illustrated in Fig. 2e and detailed in Supplementary Table 1. Key radiomic features that distinguished the subtypes include: The AP_original_firstorder_Uniformity feature measures the degree of uniformity or consistency in the distribution of pixel values within the image. This feature was lowest in Imaging Subtype 2 and highest in Imaging Subtype 3, suggesting that tumors in Subtype 2 are more heterogeneous. The AP_original_shape_SurfaceVolumeRatio feature describes the compactness and morphological complexity of the tumor in three dimensions. This feature was highest in Imaging Subtype 1, indicating that Subtype 1 tumors exhibit the most irregular morphology. A high value of PVP_original_glcm_DifferenceAverage indicates large differences in grayscale values between adjacent pixels, corresponding to high local contrast. This typically reflects a tumor with complex internal composition and high heterogeneity. This feature was highest in Imaging Subtype 2 and lowest in Imaging Subtype 3, implying that Subtype 2 tumors have more complex internal components, while those in Subtype 3 are more homogeneous.

**Table 1:** Association between imaging subtypes and clinical features

Variables	Discovery cohort (*n* = 103)				SYSUFAH validation cohort (*n* = 110)			
	Imaging type 1	Imaging type 2	Imaging type 3	*p*/adjusted *p*-values	Imaging type 1	Imaging type 2	Imaging type 3	*p*/adjusted *p*-values
	29 (28.2)	38 (36.9)	36 (34.9)		34 (30.9)	31 (28.2)	45 (40.9)	
Age median (years, range)								
	68 (50–93)	70.5 (46–86)	69.50 (31–88)	0.999/0.999	54.5 (38–75)	58 (35–75)	54 (32–76)	0.617/0.977
Sex								
Male	17 (58.6)	31 (81.6)	4 (11.1)	0.024/0.195	28 (82.4)	25 (80.6)	38 (84.4)	0.909/0.991
Female	12 (41.4)	7 (18.4)	32 (88.9)		6 (17.6)	6 (19.4)	7 (15.6)	
HBsAg								
Positive	11 (37.9)	9 (23.7)	4 (11.1)	0.040/0.195	31 (91.2)	28 (90.3)	41 (91.1)	0.991/0.991
Negative	18 (62.1)	29 (76.3)	32 (88.9)		3 (8.8)	3 (9.7)	4 (8.9)	
Evidence of cirrhosis								
No	5 (17.2)	7 (18.4)	14 (38.9)	0.065/0.195	16 (47.1)	17 (54.8)	27 (60)	0.520/0.898
Yes	24 (82.8)	31 (81.6)	22 (61.1)		18 (52.9)	14 (45.2)	18 (40)	
Child-Pugh								
A	27 (93.1)	28 (73.7)	30 (83.3)	0.180/0.360	33 (97.1)	30 (96.8)	42 (93.3)	0.672/0.982
B	2 (6.9)	9 (23.7)	6 (16.7)		1 (2.9)	1 (3.2)	3 (6.7)	
C	0 (0.0)	1 (2.6)	0 (0.0)		-	-	-	
Vascular invasion								
No	24 (82.8)	31 (81.6)	25 (69.4)	0.338/0.507	17 (50)	14 (45.2)	20 (44.4)	0.876/0.991
Yes	5 (17.2)	7 (18.4)	11 (30.6)		17 (50)	17 (54.8)	25 (55.6)	
Distant metastasis								
No	27 (93.1)	36 (94.7)	33 (91.7)	0.891/0.972	22 (64.7)	15 (48.4)	24 (53.3)	0.389/0.891
Yes	2 (6.9)	2 (5.3)	3 (8.3)		12 (35.3)	16 (51.6)	21 (46.7)	
Lymph nodes metastasis								
No	24 (82.8)	34 (89.5)	32 (88.9)	0.704/0.939	22 (64.7)	24 (77.4)	33 (73.3)	0.501/0.898
Yes	5 (17.2)	4 (10.5)	4 (11.1)		12 (35.3)	7 (22.6)	12 (26.7)	
Portal vein thrombosis								
No	25 (86.2)	33 (86.8)	29 (80.6)	0.786/0.943	20 (58.8)	18 (58.1)	25 (55.6)	0.953/0.991
Yes	4 (13.8)	5 (13.2)	7 (19.4)		14 (41.2)	13 (41.2)	20 (44.4)	
AFP median (range, ng/mL)								
	16.8 (1.8–24,478.4)	22.6 (1.4–566,530)	49.35 (1.5–67,664.9)	0.319/0.507	91.3 (0.87–275,891)	133 (2.45–2,000,000)	158 (2–1,362,263)	0.104/0.659
AFP_group								
< 400	24 (82.8)	28 (73.7)	22 (61.1)	0.148/0.355	25 (73.5)	16 (51.6)	23 (51.1)	0.092/0.659
≥ 400	5 (17.2)	10 (26.3)	14 (38.9)		9 (26.5)	15 (48.4)	22 (48.9)	
BCLC								
Stage-A	8 (27.6)	3 (7.9)	1 (2.8)	0.053/0.195	2 (5.9)	1 (3.2)	1 (2.2)	0.932/0.991
Stage-B	6 (20.7)	11 (28.9)	7 (19.4)		5 (14.7)	4 (12.9)	6 (13.3)	
Stage-C	15 (51.7)	23 (60.5)	26 (72.2)		27 (79.4)	26 (83.9)	38 (84.4)	
Stage-D	0 (0.0)	1 (2.6)	2 (5.6)		-	-	-	
ICI								
PD-1	-	-	-		32 (94.1)	28 (90.3)	41 (91.1)	0.835/0.991
PD-L1	-	-	-		2 (5.9)	3 (9.7)	4 (8.9)	
Surgical history								
No	-	-	-		18 (52.9)	20 (64.5)	35 (77.8)	0.067/0.659
Yes	-	-	-		16 (47.1)	11 (35.5)	10 (22.2)	
TACE history								
No	-	-	-		24 (70.6)	27 (87.1)	35 (77.8)	0.273/0.865
Yes	-	-	-		10 (29.4)	4 (12.9)	10 (22.2)	
HAIC history								
No	-	-	-		29 (85.3)	29 (93.5)	36 (80)	0.258/0.865
Yes	-	-	-		5 (14.7)	2 (6.5)	9 (20)	
Ablation history								
No	-	-	-		25 (73.5)	27 (87.1)	40 (88.9)	0.156/0.741
Yes	-	-	-		9 (26.5)	4 (12.9)	5 (11.1)	
Radiotherapy history								
No	-	-	-		30 (88.2)	26 (83.9)	42 (93.3)	0.422/0.891
Yes	-	-	-		4 (11.8)	5 (16.1)	3 (6.7)	
Targeted therapy history								
No	-	-	-		28 (82.4)	29 (93.5)	38 (84.4)	0.375/0.891
Yes	-	-	-		6 (17.6)	2 (6.5)	7 (15.6)	

Data are *n* (%) or median (range). Statistical evaluation was performed using ANOVA or χ^2^ (Fisher) test. For multiple comparisons, we applied the Benjamini–Hochberg correction, and the adjusted *p*-values have been listed

*AFP* Alpha-fetoprotein, *BCLC* Barcelona Clinic Liver Cancer staging, *HbsAg* Hepatitis B surface antigen, *HAIC* Hepatic artery infusion chemotherapy, *ICI* Immune checkpoint inhibitors, *PD-1* Programmed cell death 1, *PD-L1* Programmed cell death-ligand 1, *TACE* Transcatheter arterial chemoembolization

### Validated imaging subtypes as independent predictors for immunotherapy effectiveness

**Fig. 3:**
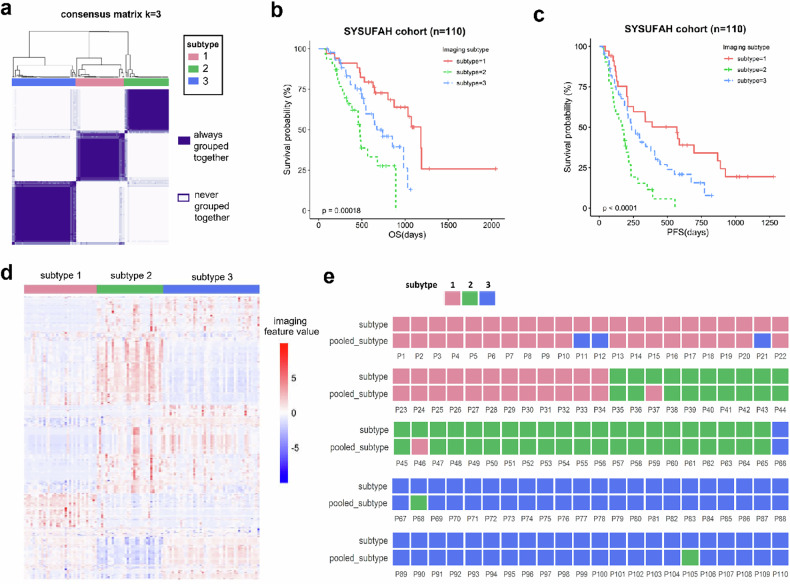
Consistent imaging subtypes were found to be associated with the effectiveness of immunotherapy. Unsupervised consensus clustering with the same clustering parameters as the discovery cohort was performed in the immunotherapy-treated SYSUFAH validation cohort. **a** A heatmap representing consensus matrices. **b** Differences in OS among imaging subtypes. **c** Differences in PFS among imaging subtypes. **d** Imaging features were visualized with a heatmap. Individual patients (columns) are grouped by imaging subtypes, and imaging features (rows) are in the same order as those displayed for the discovery cohort. **e** Image subtypes remained highly consistent whether clustering was performed directly in the SYSUFAH cohort or the pooled SYSUFAH and discovery cohorts. *p*-values were from the log-rank test. A two-sided *p* < 0.05 was considered statistically significant. OS, Overall survival; PFS, Progression-free survival; SYSUFAH, First Affiliated Hospital of Sun Yat-sen University

In the SYSUFAH image validation cohort, a total of 110 patients were included in the analysis; 77 cases were confirmed by pathology, and 33 were diagnosed *via* imaging. The same clustering procedure was applied to the immunotherapy-treated SYSUFAH image validation cohort, and three imaging subtypes were identified (Fig. 3a). The imaging subtypes had no significant association with age, sex, hepatitis B surface antigen, evidence of cirrhosis, Child-Pugh score, vascular invasion, distant metastasis, lymph nodes metastasis, portal vein thrombosis, alpha-fetoprotein, BCLC stage, ICIs received and treatment history in the SYSUFAH cohort (Table 1). We detected significant differences in OS (*p* < 0.001) and PFS (*p* < 0.0001) among the imaging subtypes in the SYSUFAH cohort (Fig. 3b, c). The 2-year OS rates for subtypes 1, 2, and 3 were 75.1%, 23.4%, and 46.6%, respectively. The 1-year PFS rates for subtypes 1, 2, and 3 were 51.6%, 11.9%, and 37.5%, respectively. Univariate and multivariate Cox regression analyses identified the imaging subtype as the sole independent predictor of survival outcomes. Specifically, for PFS, compared to Imaging Subtype 1, Subtype 2 exhibited a significantly increased risk (HR = 3.49; 95% confidence interval (CI): 1.88–6.46; *p* = 0.000), while Subtype 3 showed a more moderate elevation in risk (HR = 1.79; 95% CI: 1.02–3.14; *p* = 0.043) (Table 2). A similar trend was observed for OS: Subtype 2 was associated with a markedly higher hazard (HR = 5.54; 95% CI: 2.50–12.31; *p* = 0.000) relative to Subtype 1, and Subtype 3 also demonstrated significantly reduced survival (HR = 2.33; 95% CI: 1.11–4.89; *p* = 0.025) (Table 2). The imaging subtypes 1, 2, and 3 corresponded to favorable, poor, and intermediate immunotherapy responses.

**Table 2:** Cox analyses of the imaging subtypes and clinical factors in the SYSUFAH cohort

Predictors	PFS				OS			
	Univariate		Multivariate		Univariate		Multivariate	
	HR (95% CI)	*p*-value	HR (95% CI)	*p*-value	HR (95% CI)	*p*-value	HR (95% CI)	*p*-value
Image_subtype (2/1)	3.75 (2.04–6.9)	0.000*	3.49 (1.88–6.46)	0.000*	4.7 (2.17–10.19)	0.000*	5.54 (2.50–12.31)	0.000*
Image_subtype (3/1)	1.82 (1.04–3.19)	0.036*	1.79 (1.02–3.14)	0.043*	2.45 (1.18–5.08)	0.016*	2.33 (1.11–4.89)	0.025*
Age	0.99 (0.97–1)	0.28			1 (0.98–1.03)	0.82		
Sex (male/female)	0.88 (0.49–1.6)	0.66			0.66 (0.35–1.2)	0.2		
Surgical history (Y/N)	0.96 (0.61–1.5)	0.86			0.68 (0.38–1.2)	0.18		
TACE history (Y/N)	0.96 (0.58–1.6)	0.88			0.67 (0.35–1.3)	0.23		
HAIC history (Y/N)	1 (0.55–1.9)	0.96			0.6 (0.26–1.4)	0.24		
Ablation history (Y/N)	0.97 (0.56–1.7)	0.93			0.58 (0.28–1.2)	0.15		
Radiotherapy history (Y/N)	1.5 (0.77–2.8)	0.24			1.1 (0.5–2.4)	0.81		
Target therapy history (Y/N)	1.3 (0.7–2.5)	0.4			0.76 (0.32–1.8)	0.52		
Cirrhosis (Y/N)	0.78 (0.5–1.2)	0.26			0.9 (0.53–1.5)	0.71		
Distant metastasis (Y/N)	1.7 (1.1–2.6)	0.014*	1.54 (0.99–2.38)	0.053	1.5 (0.88–2.5)	0.14		
Lymph nodes metastasis (Y/N)	1.2 (0.72–1.9)	0.54			1.6 (0.92–2.8)	0.093		
Vascular invasion (Y/N)	1.5 (0.97–2.3)	0.066			2.3 (1.3–3.9)	0.004*	2.32 (0.95–5.67)	0.066
Portal vein thrombosis (Y/N)	1.2 (0.78–1.8)	0.42			2 (1.2–3.3)	0.011*	0.95 (0.4–2.29)	0.954
BCLC stage (B/A)	2.33 (0.51–10.61)	0.272			0.69 (0.12–3.86)	0.674		
BCLC stage (C/A)	2.78 (0.68–11.4)	0.157			1.87 (0.45–7.83)	0.391		
Child-Pugh (B/A)	1.2 (0.44–3.3)	0.72			1.9 (0.58–6)	0.3		
ICI (PD-L1/PD-1)	0.5 (0.2–1.2)	0.13			0.56 (0.17–1.8)	0.33		
AFP (≥ 400/< 400)	1.2 (0.78–1.8)	0.41			2 (1.2–3.4)	0.009*	1.37 (0.72–2.6)	0.345

Statistical evaluation was performed using the Cox proportional hazard model (* *p* < 0.05)

*AFP* Alpha-fetoprotein, *BCLC* Barcelona Clinic Liver Cancer staging, *HAIC* Hepatic artery infusion chemotherapy, *ICI* Immune checkpoint inhibitors, *OS* Overall survival, *PFS* Progression-free survival, *PD-1* Programmed cell death 1, *PD-L1* Programmed cell death-ligand 1, *SYSUFAH* First Affiliated Hospital of Sun Yat-sen University, *TACE* Transcatheter arterial chemoembolization

The consistency of the three imaging subtypes in the validation cohort compared to those in the discovery cohort is crucial. The imaging feature heatmap indicated that the profiles of subtypes are consistent (Fig. 3d). The corresponding IGP values of imaging subtypes 1, 2, and 3 between the discovery and validation cohorts are 83.8%, 93.5%, and 95.2%, respectively. To further verify the consistency of these imaging subtypes, we pooled the patients from both the validation and discovery cohorts and performed the same clustering process. We compared the clustering results to those from direct clustering of the validation cohort. Ultimately, we discovered that only 7 of the 110 patients had inconsistent subtypes (Fig. 3e). Moreover, through principal component analysis, we found that the principal components of imaging features showed no significant differences between the discovery and validation cohorts across Imaging Subtypes 1, 2, and 3 (Figs. S1–3). This indicates that our clustering method shows great potential for clinical practice.

### Validated imaging subtypes were significantly associated with OS in the gene validation cohorts

**Fig. 4:**
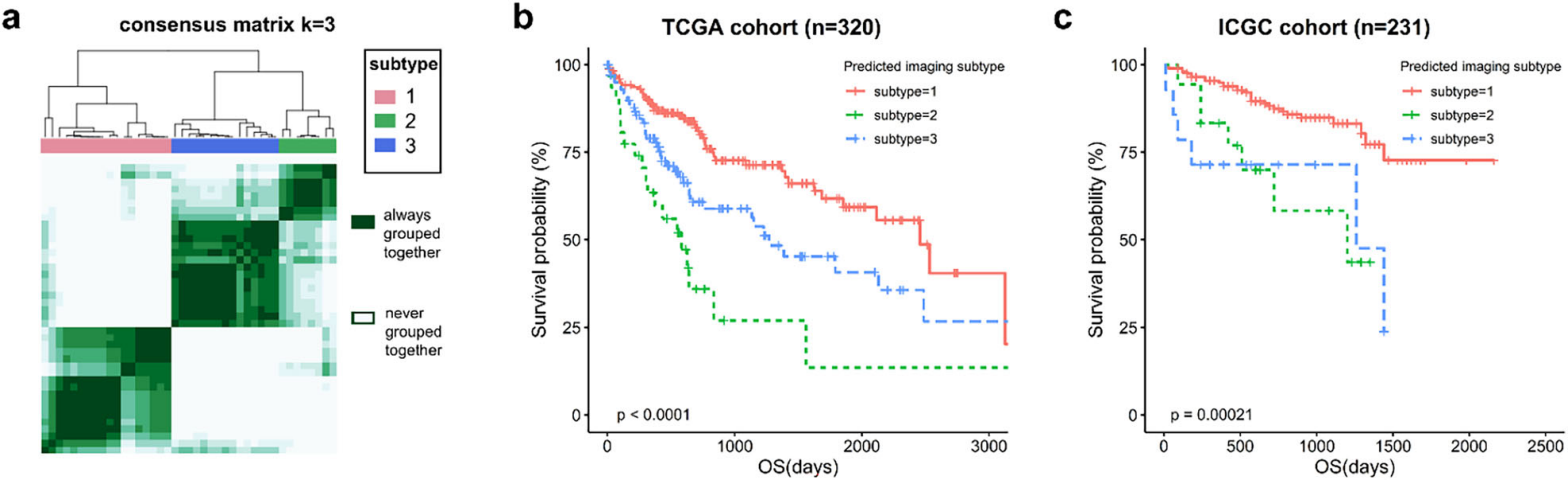
Imaging subtypes were validated and significantly associated with OS in the gene validation cohorts. Unsupervised consensus clustering with the same clustering parameters as the discovery cohort was performed in the TCGA-LIHC imaging validation cohort. **a** A heatmap representing consensus matrices. **b** Differences in OS among imaging subtypes in the TCGA gene validation cohort. **c** Differences in OS among imaging subtypes in the ICGC gene validation cohort. *p*-values were from the Log-rank test. A two-sided *p* < 0.05 was considered statistically significant. ICGC, International Cancer Genome Consortium; TCGA-LIHC, The Cancer Genome Atlas Liver Hepatocellular Carcinoma; TCGA, The Cancer Genome Atlas

The same clustering procedure was applied to the TCGA-LIHC image validation cohort, and three imaging subtypes were identified (Fig. 4a). In the TCGA-LIHC image validation cohort, imaging subtypes 2 and 3 displayed a high level of consistency, with corresponding IGP values of 93.3% and 93.8%, respectively. The imaging subtype 1 showed more inter-tumor phenotypic heterogeneity, with a lower IGP value of 60.0%. Based on the imaging subtype labels and RNA-seq data of the TCGA-LIHC image validation cohort, we trained a nearest shrunken centroid classifier to predict imaging subtypes in the gene validation cohorts. The accuracy of the gene-based classifier in predicting imaging subtypes 1, 2, and 3 was 94.4%, 80%, and 75%, respectively (Supplementary Table S2). We applied the gene-based classifier to assign each patient in the ICGC-LIRI-JP and TCGA-LIHC gene validation cohorts into one of the three imaging subtypes. Predicted imaging subtypes exhibited significant differences in OS in both TCGA-LIHC (*p* < 0.001, Fig. 4b) and ICGC-LIRI-JP (*p* < 0.001, Fig. 4c) gene validation cohorts. The average 2-year OS rates were 83.7%, 47.2%, and 62.1% for subtypes 1, 2, and 3, respectively.

### Imaging subtypes revealed underlying immune mechanisms

**Fig. 5:**
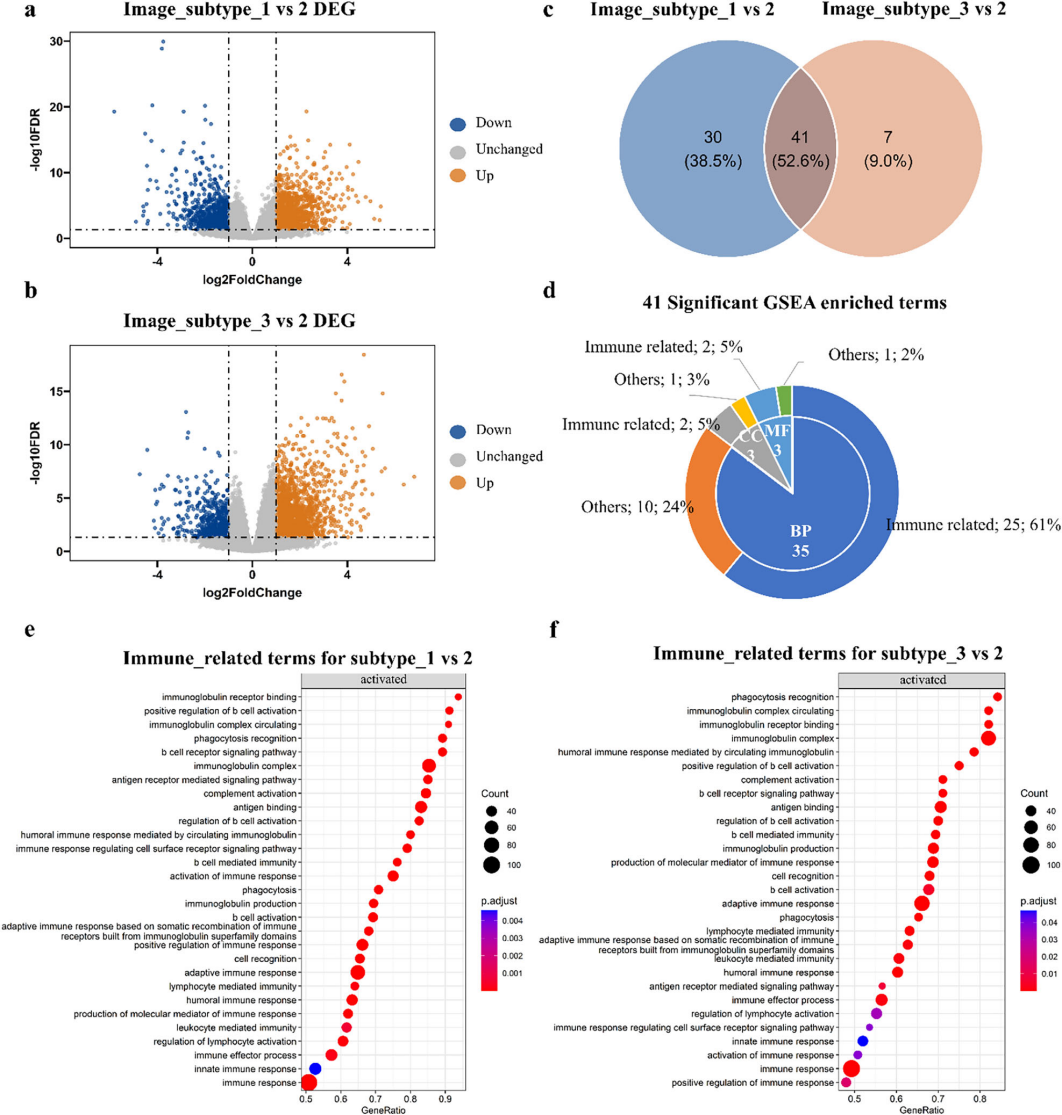
Imaging subtypes revealed underlying immune mechanisms. **a** Significant differential gene expression (Imaging subtype 1 *versus* 2). **b** Significant differential gene expression (Imaging subtype 3 *versus* 2). **c** Venn diagram displaying 41 enriched terms in both imaging subtypes 1 and 3. **d** 71% of these terms are immune-related. **e**, **f** Dotplot of the enriched immune-related terms. DEG and GSEA were conducted using the R package “DESeq2” and “clusterProfiler.” Genes with FDR 0.05 and |FC| > 2 were significantly differentially expressed. Pathways with adjusted *p* < 0.05 and FDR < 0.05 were identified as significantly enriched. BP, Biological process; CC, Cellular component; DEG, Differential expression genes; FDR, False discovery rate; FC, Fold change; GSEA, Gene set enrichment analysis; MF, Molecular function

Differential gene expression analysis revealed 1,019 downregulated and 1,282 upregulated genes in imaging subtype 1 compared to subtype 2 (Fig. 5a). Meanwhile, 621 genes were downregulated and 2,070 genes were upregulated in imaging subtype 3 (Fig. 5b). Based on these genes, GSEA identified 71 enriched terms in imaging subtype 1 and 48 in subtype 3 (Supplementary Tables S3 and S4, Fig. 5c). Forty-one terms were enriched in both imaging subtypes 1 and 3, with 71% being immune-related (Fig. 5c, d). The enriched immune-related terms were displayed in Fig. 5e, f. In comparison to imaging subtype 2, several immune processes enriched in imaging subtypes 1 and 3 were shown in Supplementary Fig. S4. Imaging subtype 1 showed significant activation of B-cell, lymphocyte activation, immune response activation, and innate immune response (Supplementary Fig. S4a–d). In addition, imaging subtype 3 exhibited significant activation of B-cell, lymphocyte-mediated immunity, immune response activation, and innate immune response (Supplementary Fig. S4e–h). In conclusion, the molecular mechanisms underlying imaging subtypes involve both innate and adaptive immune activation, and the imaging subtypes could reveal distinct immune mechanisms.

### Imaging subtypes significantly associated with tumor immune microenvironment

**Fig. 6:**
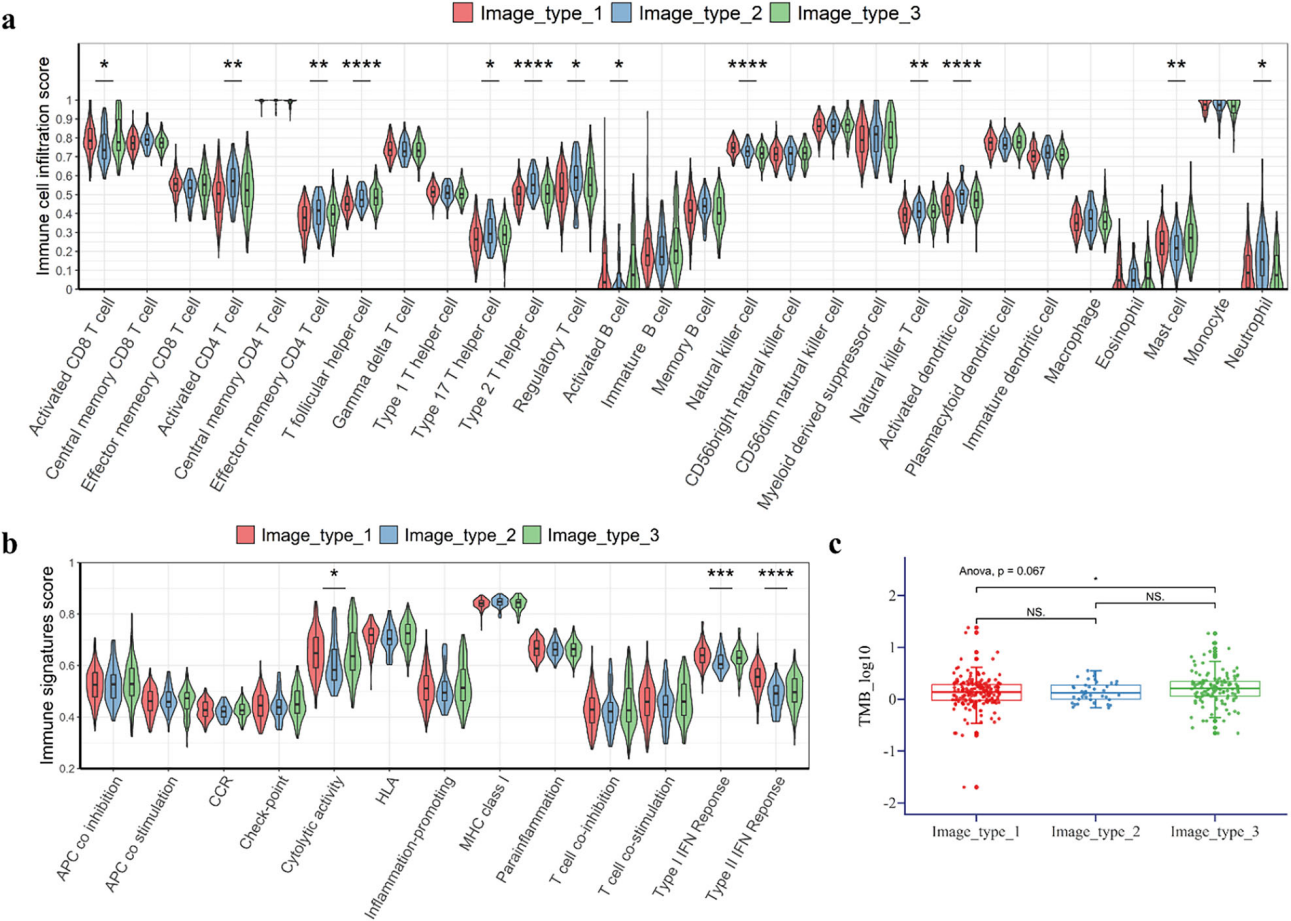
Imaging subtypes were correlated with tumor immune microenvironment features. **a** Correlations between imaging subtypes and infiltration of 28 immune cells. **b** Correlations between imaging subtypes and 13 immune signature scores. **c** Association between TMB and imaging subtypes. GSEA was conducted using the R package “clusterProfiler.” Pathways with adjusted *p* < 0.05 and FDR < 0.05 were identified as significantly enriched. Statistical evaluation was performed using ANOVA, Kruskal–Wallis test, or Student’s *t*-test (* *p* ≤ 0.05; ** *p* ≤ 0.01; *** *p* ≤ 0.001; **** *p* ≤ 0.0001; ns: *p* > 0.05). GSEA, Gene set enrichment analysis; NES, Normalized enrichment score

Since the imaging subtypes could reveal distinct underlying immune mechanisms, we conducted a detailed analysis of correlations between imaging subtypes and tumor immune microenvironments. In Fig. 6, we presented the correlations between imaging subtypes and the infiltration of 28 immune cells along with 13 immune signature scores. Imaging subtype 2 with poor immunotherapy responses had the lowest infiltration of activated CD8+ T cells, activated B-cells, and mast cells (Fig. 6a). On the other hand, it had the highest infiltration of Th17 helper cells, Th2 helper cells, regulatory T cells, natural killer cells, and neutrophils. Cytolytic activity was significantly lower in imaging subtype 2 compared to other subtypes (Fig. 6b). Type I and II interferon (IFN) responses were significantly downregulated in imaging subtype 2 and upregulated in imaging subtype 1 (Fig. 6b). We also analyzed the correlation between TMB and imaging subtypes. Although no significant differences were found, it can be seen that imaging subtype 2 had the lowest TMB level (Fig. 6c).

## Discussion

In this study, we identified three non-invasive imaging subtypes of HCC that are significantly associated with OS and predictive of immunotherapy response. These subtypes could reflect distinct immune microenvironments characterized by variations in B-cell activation, lymphocyte-mediated immunity, and innate immune responses.

Sia et al identified the immune class characterized by high cytolytic activity and fewer chromosomal aberrations, resembling melanomas responsive to immunotherapies [31]. Similarly, Montironi et al defined an “inflamed class” characterized by elevated interferon signaling, cytolytic activity, immune-effector cytokine expression, and a diverse T-cell repertoire, which correlates with immunotherapy response [32]. Molecular analyses from the GO30140 and IMbrave150 trials in HCC patients linked improved outcomes after immunotherapy to high CD274 expression, a T-effector signature, and intratumoral CD8⁺ T-cell density, whereas a high Treg/Teff (regulatory T cell/ effector T cell) ratio was associated with reduced benefit [33]. In our study, imaging subtype 1—exhibiting high levels of activated CD8⁺ T cells, cytolytic activity, interferon signaling, and immune activation—showed the most favorable response to immunotherapy. In contrast, subtype 2, defined by a high Treg/Teff ratio, had the poorest response. These findings align with previously established molecular features of immunotherapy-responsive subtypes.

Imaging subtypes 1 and 3 exhibited increased B-cell activation compared to subtype 2 (Supplementary Fig. S4a, e, Fig. 6a). Previous studies have linked high B-cell infiltration to improved survival [34], potentially through multiple mechanisms: secretion of tumor-specific antibodies, antigen presentation to CD4⁺ and CD8⁺ T cells, NK cell stimulation, and release of T-cell activating factors [35]. Spatial transcriptomics in HCC patients responding to cabozantinib and PD-1 inhibition revealed enriched cancer-immune interactions dominated by B-cell activity [36]. These findings underscore the need to further investigate B-cells’ role in the tumor microenvironment and their potential as predictors of immunotherapy response in HCC.

High levels of tumor-associated neutrophils have been associated with tumor progression and poor prognosis [37]. Activation of neutrophil-related pathways correlates with disease progression following pembrolizumab treatment in HCC [38]. Correspondingly, imaging subtype 2, which showed the worst survival, exhibited the most neutrophil infiltration. Interferons (IFNs), including type I (*e.g*., IFN-α, IFN-β) and type II (IFN-γ), exert antiproliferative, antiviral, and immunomodulatory effects [39]. Responders to anti-PD-1 therapy in HCC demonstrate upregulated IFN-α [40] and IFN-γ [41] signatures, and type I IFN activation triggers antitumor immunity and enhances anti-PD-1 efficacy [42, 43]. In our study, both type I and II IFN responses were significantly downregulated in subtype 2 and upregulated in subtype 1 (Fig. 6b), suggesting that imaging subtypes may enable non-invasive monitoring of underlying molecular activity, offering a better-tolerated alternative to invasive biopsies.

In this study, we demonstrate that imaging subtypes can predict prognosis and immunotherapy response in HCC patients and are significantly associated with the tumor microenvironment. Several limitations should be noted. First, although preprocessing steps were applied to reduce scanner-related variability, no formal harmonization approach (*e.g*., ComBat) was used. Multicenter radiomics features are known to be sensitive to differences in scanner manufacturer, reconstruction kernel, and acquisition parameters, which can alter feature distributions independent of tumor biology. Consequently, residual inter-scanner effects may still influence clustering outcomes and limit the generalizability of imaging subtypes. Future studies should consider incorporating cross-scanner harmonization to further enhance reproducibility and ensure that observed imaging subtypes primarily reflect biological rather than technical variation. Second, intrinsic subtype exploration assigned equal weight to all quantitative imaging features without weighting analysis; future studies should identify the most discriminative features. Third, although subtype consistency between discovery and validation cohorts was high, inter-observer variability remains a potential concern and needs future studies. Fourth, the gene-based classifier showed relatively low accuracy in predicting imaging subtype 3, possibly due to tumor heterogeneity and cross-population genetic differences. Fifth, most cases of HCC are diagnosed at an advanced stage, making surgical intervention no longer an option [5]. The histological diagnosis of HCC is seldom necessary now, as non-invasive methods are preferred [8]. Therefore, tumor tissues required for identifying histopathological characteristics were not always available. This highlights that imaging subtypes hold crucial value in predicting immunotherapy efficacy. However, further research is necessary to examine the relationship between imaging subtypes and histopathological characteristics and address the limitation of relying solely on sequencing data for evaluating the associations between imaging features and genetic characteristics. Finally, our findings require validation in larger prospective HCC cohorts, and additional *in vivo* and *in vitro* experiments are necessary to establish a direct link between imaging subtypes and the tumor microenvironment.

In conclusion, three CT-based imaging subtypes were identified from arterial phase and portal venous phase scans. These imaging subtypes can predict overall survival and immunotherapy response, and could additionally reflect molecular immune patterns, representing a valuable clinical biomarker for HCC immunotherapy efficacy.

## Supplementary information

**Additional File :** **Fig. S1:**Comparison of principal components between the discovery and validation cohorts in imaging subtype 1. The principal components of imaging features showed no significant differences among imaging subtype 1 in both the discovery cohort and the validation cohort. A t-test was used when the data followed a normal distribution; otherwise, a non-parametric test (Mann-Whitney U test) was employed to compare the differences in principal components. A p-value < 0.05 was considered statistically significant. **Fig. S2:** Comparison of principal components between the discovery and validation cohorts in imaging subtype 2.The principal components of imaging features showed no significant differences among imaging subtype 2 in both the discovery cohort and the validation cohort. A t-test was used when the data followed a normal distribution; otherwise, a non-parametric test (Mann-Whitney U test) was employed to compare the differences in principal components. A p-value < 0.05 was considered statistically significant. **Fig. S3:** Comparison of principal components between the discovery and validation cohorts in imaging subtype 3. The principal components of imaging features showed no significant differences among imaging subtype 3 in both the discovery cohort and the validation cohort. A t-test was used when the data followed a normal distribution; otherwise, a non-parametric test (Mann-Whitney U test) was employed to compare the differences in principal components. A p-value < 0.05 was considered statistically significant. Fig. S4: Several innate and adaptive immune processes associated with imaging subtypes determined by GSEA. Compared to imaging subtype 2, imaging subtype 1 exhibited significant activation of B cells (**a**), lymphocytes (**b**), immune response (**c**), and innate immune response (**d**). And imaging subtype 3 displayed significant activation of B cells (**e**), lymphocyte-mediated immunity (**f**), immune response (**g**), and innate immune response (**h**). GSEA was conducted using the R package “clusterProfiler”. Pathways with adjusted p<0.05 and FDR<0.05 were identified as significantly enriched. Table S1. Categories of quantitative imaging features extracted from the AP or PVP images. **Table S2.** Confusion matrices of the gene classifier. **Table S3.** Significant GSEA enriched immune-related terms based on differential genes of imaging subtype 1 vs 2. **Table S4.** Significant GSEA enriched terms based on differential genes of imaging subtype 3 vs 2.

## Data Availability

The publicly available data utilized in this study were obtained from public databases (https://www.cancerimagingarchive.net/, https://portal.gdc.cancer.gov/projects/TCGA-LIHC, https://dcc.icgc.org/projects/LIRI-JP). The images and clinical data collected at our center are not publicly available because they contain private patient information. Derived result data supporting the findings of this study are available upon reasonable request from the corresponding authors.

## Abbreviations

BCLC: Barcelona Clinic Liver Cancer
CI: Confidence interval
FDR: False discovery rate
GSEA: Gene set enrichment analysis
HCC: Hepatocellular carcinoma
ICGC: International Cancer Genome Consortium
ICIs: Immune checkpoint inhibitors
IFN: Interferon
IGP: In-group proportion
OS: Overall survival
PD-L1: Programmed death-ligand 1
PFS: Progression-free survival
SYSUFAH: First Affiliated Hospital of Sun Yat-sen University
TCGA: The Cancer Genome Atlas
TCGA-LIHC: TCGA-liver hepatocellular carcinoma
TMB: Tumor mutational burden
