# Supplementary material for: Clustering of quantitative CT features identifies HCC subtypes with distinct prognosis and immune signatures
Source: Eur Radiol Exp. 2026 May 27;10:75. doi: 10.1186/s41747-026-00730-1 (PMC13216361; doi:10.1186/s41747-026-00730-1)
Supplement: Supplementary file 1 — Additional File : Fig. S1:Comparison of principal components between the discovery and validation cohorts in imaging subtype 1. The principal components of imaging features showed no significant differences among imaging subtype 1 in both the discovery cohort and the validation cohort. A t-test was used when the data followed a normal distribution; otherwise, a non-parametric test (Mann-Whitney U test) was employed to compare the differences in principal components. A p-value < 0.05 was considered statistically significant. Fig. S2: Comparison of principal components between the discovery and validation cohorts in imaging subtype 2.The principal components of imaging features showed no significant differences among imaging subtype 2 in both the discovery cohort and the validation cohort. A t-test was used when the data followed a normal distribution; otherwise, a non-parametric test (Mann-Whitney U test) was employed to compare the differences in principal components. A p-value < 0.05 was considered statistically significant. Fig. S3: Comparison of principal components between the discovery and validation cohorts in imaging subtype 3. The principal components of imaging features showed no significant differences among imaging subtype 3 in both the discovery cohort and the validation cohort. A t-test was used when the data followed a normal distribution; otherwise, a non-parametric test (Mann-Whitney U test) was employed to compare the differences in principal components. A p-value < 0.05 was considered statistically significant. Fig. S4: Several innate and adaptive immune processes associated with imaging subtypes determined by GSEA. Compared to imaging subtype 2, imaging subtype 1 exhibited significant activation of B cells (a), lymphocytes (b), immune response (c), and innate immune response (d). And imaging subtype 3 displayed significant activation of B cells (e), lymphocyte-mediated immunity (f), immune response (g), and innate immune response (h). GSEA was con [file 41747_2026_730_MOESM1_ESM.pdf]

Clustering of quantitative CT features identifies HCC subtypes with distinct prognosis and immune signatures

ELECTRONIC SUPPLEMENTARY MATERIAL

Table of content

CT acquisition protocols

Fig. S1: Comparison of principal components between the discovery and validation cohorts in imaging subtype 1.

Fig. S2: Comparison of principal components between the discovery and validation cohorts in imaging subtype 2.

Fig. S3: Comparison of principal components between the discovery and validation cohorts in imaging subtype 3.

Fig. S4: Several innate and adaptive immune processes associated with imaging subtypes determined by GSEA.

Table S1: Categories of quantitative imaging features extracted from the AP or PVP images

Table S2: Confusion matrices of the gene classifier.

Table S3. Significant GSEA enriched immune-related terms based on differential genes of imaging subtype 1 vs 2

Table S4. Significant GSEA enriched terms based on differential genes of imaging subtype 3 vs 2

CT acquisition protocols

For the images downloaded from the public database, the CT acquisition protocols have been described in detail in the published article[1]. All patients underwent contrast-enhanced CT of the abdomen, with liver protocol on 16–, or 64–detector row CT scanners (LightSpeed; GE Healthcare, Waukesha, WI, USA). A pre-contrast scan was obtained, followed by an arterial phase scan 17 seconds after peak enhancement (using bolus tracking) of the aorta after injection of contrast medium. The porto-venous phase was scanned at 60 seconds. Images were acquired with the following scanner parameters: CT tube voltage of 120–140 KVp; Tube current of 150–630 mA; slice thickness of 0.63–5 mm; Pitch of 0.9–0.98; revolution time of 0.40–0.80 seconds; table speed of 18.75–39.38 mm/gantry rotation and field of view of 360–460 mm. The injection rate of contrast medium was 3–5 ml/sec. Standard image reconstruction algorithm was used in all cases[1]. The CT acquisition parameters at our institution were as follows: Images were obtained using either a 64-detector row (Aquilion CXL, Toshiba Medical Systems, Tokyo, Japan) or 320-detector row CT scanner (Aquilion One, Toshiba Medical Systems, Tokyo, Japan). Contrast-enhanced scans were performed at 35 seconds (arterial phase) and 65 seconds (portal venous phase) after intravenous administration of the contrast agent (Ultravist 300, Bayer Schering Pharma, Berlin, Germany; dose: 1.5 mL/kg body weight; injection rate: 3-4 mL/s). The scanning protocol was set as follows: tube voltage, 120 kV; tube current, 250 mA; and slice thicknesses, 1 mm and 10 mm.

References:

1. Moawad, A.W., et al. Multimodality annotated hepatocellular carcinoma data set including pre- and post-TACE with imaging segmentation. Sci Data 10, 33 (2023).

Fig. S1: **Comparison of principal components between the discovery and validation cohorts in imaging subtype 1.**

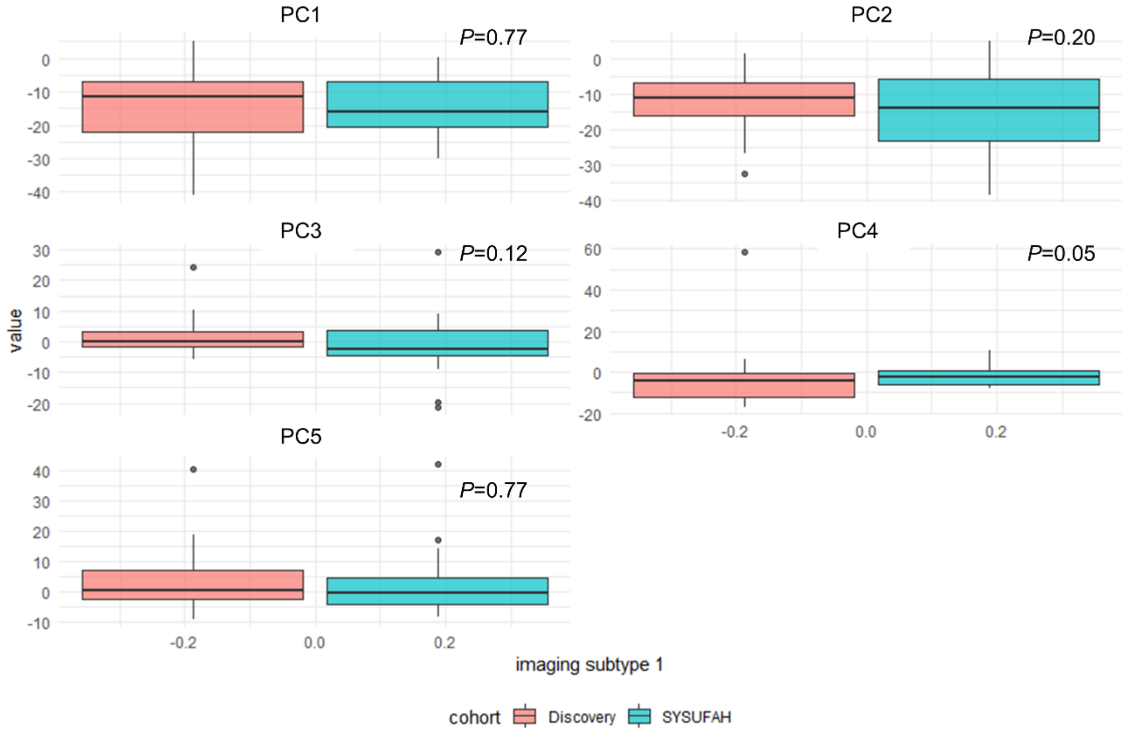

The principal components of imaging features showed no significant differences among imaging subtype 1 in both the discovery cohort and the validation cohort. A t-test was used when the data followed a normal distribution; otherwise, a non-parametric test (Mann-Whitney U test) was employed to compare the differences in principal components. A p-value < 0.05 was considered statistically significant. PC: principal components. SYSUFAH: First Affiliated Hospital of Sun Yat-sen University.

Fig. S2: **Comparison of principal components between the discovery and validation cohorts in imaging subtype 2.**

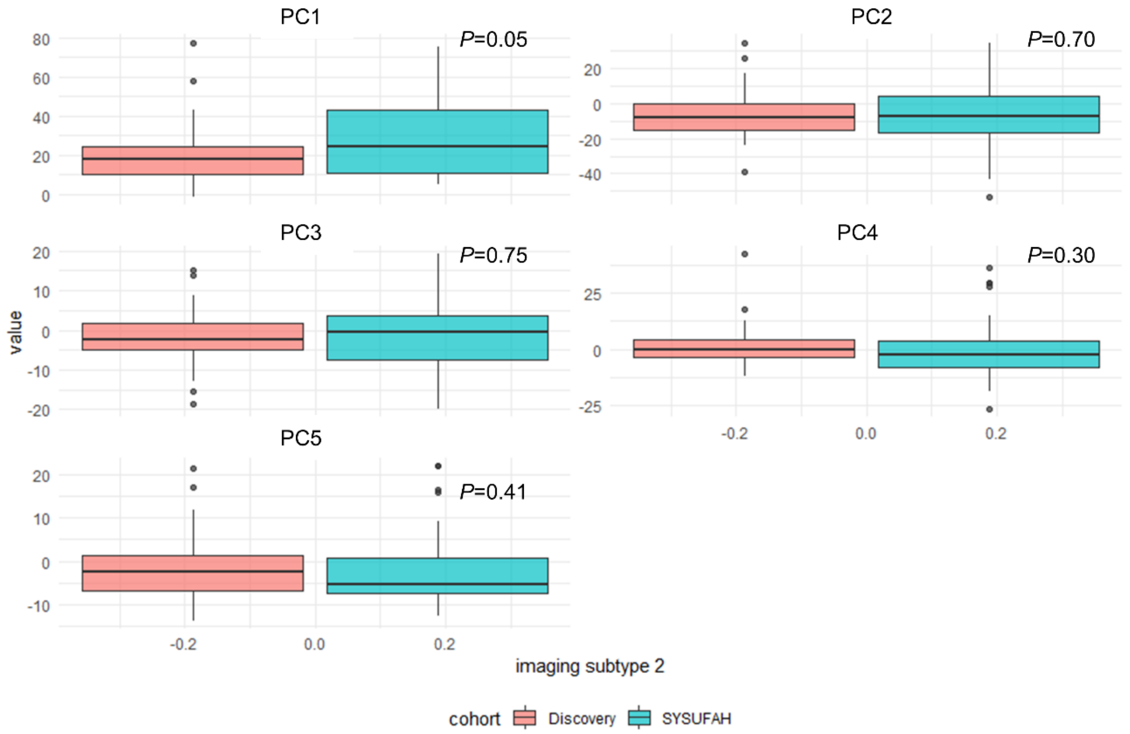

The principal components of imaging features showed no significant differences among imaging subtype 2 in both the discovery cohort and the validation cohort. A t-test was used when the data followed a normal distribution; otherwise, a non-parametric test (Mann-Whitney U test) was employed to compare the differences in principal components. A p-value < 0.05 was considered statistically significant. PC: principal components. SYSUFAH: First Affiliated Hospital of Sun Yat-sen University.

Fig. S3: **Comparison of principal components between the discovery and validation cohorts in imaging subtype 3.**

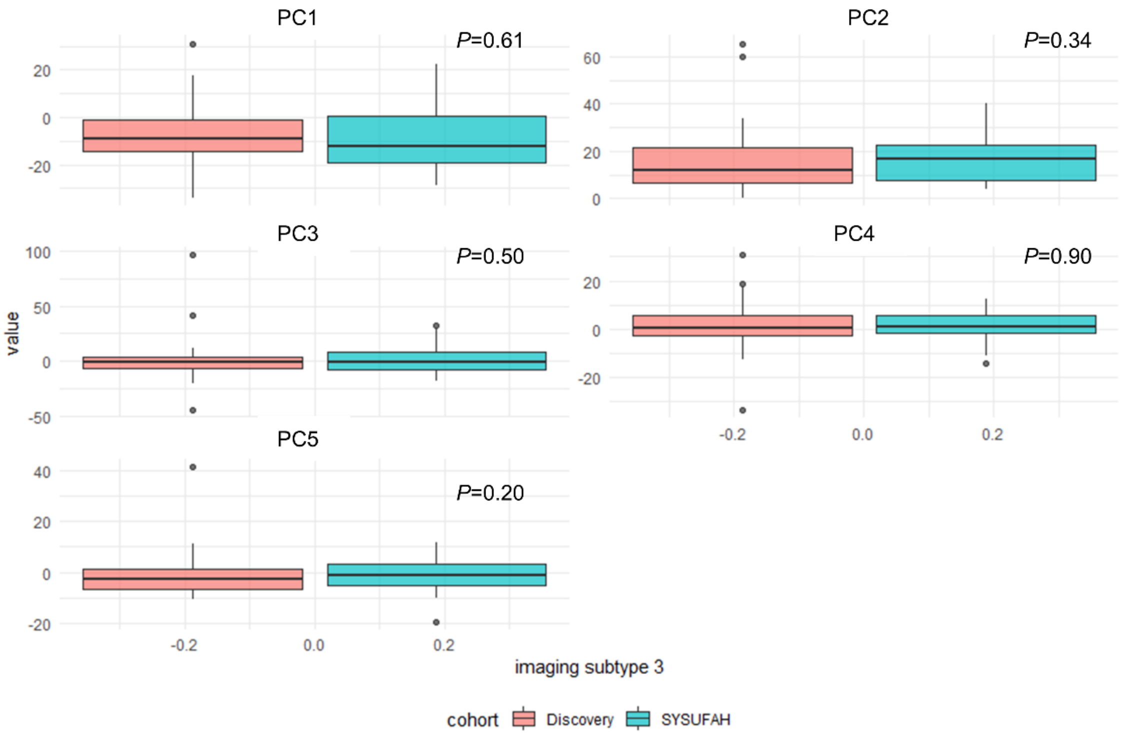

The principal components of imaging features showed no significant differences among imaging subtype 3 in both the discovery cohort and the validation cohort. A t-test was used when the data followed a normal distribution; otherwise, a non-parametric test (Mann-Whitney U test) was employed to compare the differences in principal components. A p-value < 0.05 was considered statistically significant. PC: principal components. SYSUFAH: First Affiliated Hospital of Sun Yat-sen University.

Fig. S4: **Several innate and adaptive immune processes associated with imaging subtypes determined by GSEA.**

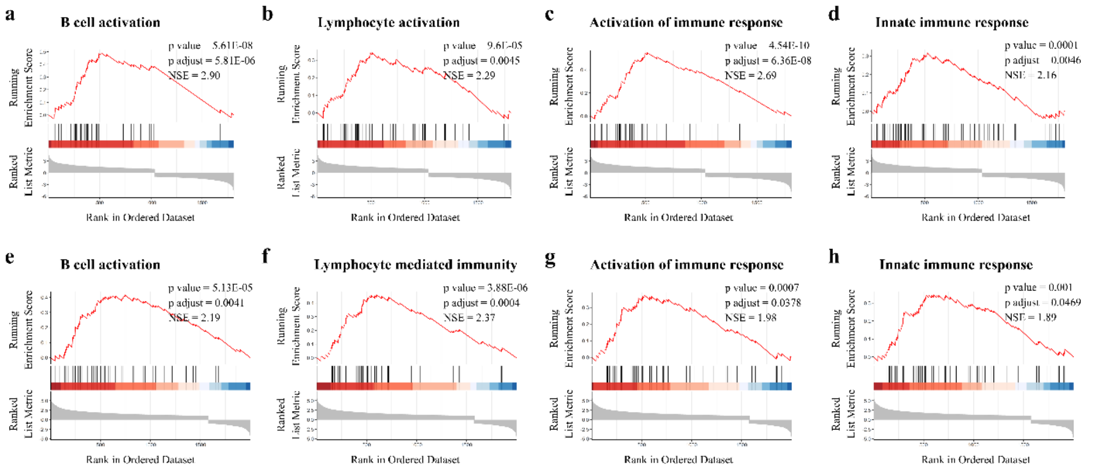

Compared to imaging subtype 2, imaging subtype 1 exhibited significant activation of B cells (a), lymphocytes (b), immune response (c), and innate immune response (d). And imaging subtype 3 displayed significant activation of B cells (e), lymphocyte-mediated immunity (f), immune response (g), and innate immune response (h). GSEA was conducted using the R package “clusterProfiler”. Pathways with adjusted p<0.05 and FDR<0.05 were identified as significantly enriched. GSEA: gene set enrichment analysis; NES: normalized enrichment score; FDR: false discovery rate.

Table S1. Categories of quantitative imaging features extracted from the AP or PVP images

Features categories	Feature number
shape	14
first-order statistics	18
gray level cooccurence matrix	24
gray level dependence matrix	14
gray level run length matrix	16
gray level size zone matrix	16
neighboring gray tone difference matrix	5
wavelet features	744
total	851

AP: arterial phase, PVP: portal venous phase

Table S2. Confusion matrices of the gene classifier.

	predicted subtype 1	predicted subtype 2	predicted subtype 3
actually subtype 1	17	1	0
actually subtype 2	3	12	0
actually subtype 3	2	0	6

Table S3. Significant GSEA enriched immune-related terms based on differential genes of imaging subtype 1 vs 2

Terms	setSize	EnrichmentScore	NES	pvalue	p.adjust	qvalue
immunoglobulin_complex	75	0.655167725	4.28071	1E-10	1.98E-08	1.93E-08
antigen_binding	59	0.612983421	3.783095	1E-10	1.98E-08	1.93E-08
complement_activation	45	0.632957812	3.552073	1E-10	1.98E-08	1.93E-08
adaptive_immune_response	111	0.498893024	3.551987	1E-10	1.98E-08	1.93E-08
phagocytosis_recognition	37	0.670943397	3.501924	1E-10	1.98E-08	1.93E-08
immunoglobulin_receptor_binding	32	0.698347262	3.451849	1E-10	1.98E-08	1.93E-08
b_cell_receptor_signaling_pathway	37	0.660282985	3.446283	1E-10	1.98E-08	1.93E-08
positive_regulation_of_b_cell_activation	34	0.683348721	3.446109	1E-10	1.98E-08	1.93E-08
immunoglobulin_complex_circulating	33	0.678617626	3.399279	1E-10	1.98E-08	1.93E-08
antigen_receptor_mediated_signaling_pathway	40	0.615755451	3.328411	1E-10	1.98E-08	1.93E-08
activation_of_immune_response	56	0.546890283	3.285855	1E-10	1.98E-08	1.93E-08
immunoglobulin_production	49	0.563084755	3.266665	1E-10	1.98E-08	1.93E-08
regulation_of_b_cell_activation	40	0.603860986	3.264117	1.36E-10	2.49E-08	2.42E-08
humoral_immune_response_mediated_by_circulating_immunoglobulin	40	0.598353352	3.234346	2.37E-10	3.76E-08	3.65E-08
positive_regulation_of_immune_response	71	0.477283412	3.081385	3.39E-10	5.04E-08	4.89E-08
immune_response	203	0.3334475	2.692403	4.54E-10	6.36E-08	6.18E-08
immune_response_regulating_cell_surface_receptor_sig	43	0.571653686	3.153398	6.74E-10	8.91E-08	8.65E-08

naling_pathway						
b_cell_mediated_immunity	42	0.571020981	3.148067	1.02E-09	1.28E-07	1.25E-07
production_of_molecular_mediator_of_immune_response	58	0.501193899	3.07232	2.06E-09	2.45E-07	2.38E-07
adaptive_immune_response_based_on_somatic_recombination_of_immune_receptors_built_from_immunoglobulin_superfamily_domains	50	0.501078031	2.924691	3.56E-08	4.03E-06	3.92E-06
b_cell_activation	52	0.491514834	2.90152	5.61E-08	5.81E-06	5.64E-06
cell_recognition	55	0.478670433	2.883637	1.03E-07	1.02E-05	9.9E-06
Phagocytosis	48	0.494384358	2.860457	2.56E-07	2.34E-05	2.27E-05
humoral_immune_response	68	0.436664177	2.778055	2.65E-07	2.34E-05	2.27E-05
regulation_of_lymphocyte_activation	66	0.416386448	2.637293	6.83E-07	5.42E-05	5.26E-05
lymphocyte_mediated_immunity	50	0.458008385	2.673302	1.33E-06	9.62E-05	9.35E-05
immune_effector_process	82	0.387098955	2.589501	2.71E-06	0.00019	0.000184
leukocyte_mediated_immunity	60	0.412947683	2.547073	9.53E-06	0.000613	0.000595
lymphocyte_activation	82	0.34195601	2.287517	9.6E-05	0.00448	0.004354
innate_immune_response	93	0.317530783	2.16448	0.0001	0.004584	0.004454

GSEA was conducted using the R package “clusterProfiler”. Pathways with adjusted p<0.05 and FDR<0.05 were identified as significantly enriched. GSEA: gene set enrichment analysis; NES: normalized enrichment score; FDR: false discovery rate.

Table S4. Significant GSEA enriched terms based on differential genes of imaging subtype 3 vs 2

Terms	setSize	EnrichmentScore	NES	pvalue	p.adjust	qvalue
immunoglobulin_complex	89	0.59559	3.384327	1E-10	8.21E-08	7.51E-08
antigen_binding	68	0.535407	2.860294	1.07E-10	8.21E-08	7.51E-08
adaptive_immune_response	121	0.475881	2.83591	1E-10	8.21E-08	7.51E-08
production_of_molecular_mediator_of_immune_response	64	0.531727	2.818565	9.11E-10	5.24E-07	4.79E-07
immunoglobulin_production	61	0.530669	2.773366	2.5E-09	1.15E-06	1.05E-06
phagocytosis_recognition	38	0.602637	2.813427	4.32E-09	1.66E-06	1.51E-06
cell_recognition	53	0.527096	2.65604	1.29E-08	4.24E-06	3.87E-06
immunoglobulin_complex_circulating	39	0.587833	2.768166	1.89E-08	4.82E-06	4.41E-06
immunoglobulin_receptor_binding	39	0.587833	2.768166	1.89E-08	4.82E-06	4.41E-06
positive_regulation_of_b_cell_activation	44	0.551223	2.671523	1.07E-07	2.25E-05	2.06E-05
humoral_immune_response_mediated_by_circulating_immunoglobulin	42	0.559304	2.671789	1.45E-07	2.77E-05	2.53E-05
complement_activation	45	0.544565	2.654213	1.79E-07	3.17E-05	2.9E-05
regulation_of_b_cell_activation	50	0.508158	2.532535	5.28E-07	8.68E-05	7.93E-05
Phagocytosis	49	0.505522	2.512299	6.18E-07	9.48E-05	8.66E-05
humoral_immune_response	68	0.454481	2.427966	7.33E-07	0.000105	9.63E-05
b_cell_mediated_immunity	49	0.501789	2.493751	8.51E-07	0.000115	0.000105
immune_response	205	0.323079	2.059902	2.42E-06	0.000293	0.000268

b_cell_receptor_signaling_pathway	45	0.510449	2.487933	3E-06	0.000346	0.000316
lymphocyte_mediated_immunity	57	0.458571	2.373425	3.88E-06	0.000425	0.000389
adaptive_immune_response_based_on_somatic_recombination_of_immune_receptors_built_from_immunoglobulin_superfamily_domains	59	0.440564	2.285601	1.03E-05	0.001077	0.000985
leukocyte_mediated_immunity	66	0.429551	2.296573	1.4E-05	0.001243	0.001136
immune_effector_process	85	0.397242	2.236223	1.37E-05	0.001243	0.001136
b_cell_activation	62	0.416588	2.185951	5.13E-05	0.004137	0.003781
antigen_receptor_mediated_signaling_pathway	53	0.422146	2.127198	9.6E-05	0.00735	0.006718
positive_regulation_of_immune_response	75	0.365472	1.999122	0.000262	0.017196	0.015718
regulation_of_lymphocyte_activation	76	0.36132	1.982084	0.000547	0.033094	0.030249
immune_response_regulating_cell_surface_receptor_signaling_pathway	56	0.401493	2.061308	0.000671	0.037709	0.034467
activation_of_immune_response	63	0.375248	1.980449	0.000706	0.0378	0.03455
innate_immune_response	75	0.345712	1.891034	0.000958	0.046905	0.042872

GSEA was conducted using the R package “clusterProfiler”. Pathways with adjusted p<0.05 and FDR<0.05 were identified as significantly enriched.

GSEA: gene set enrichment analysis; NES: normalized enrichment score; FDR: false discovery rate.
